# A proof-of-concept study evaluating the effect of ADX10059, a metabotropic glutamate receptor-5 negative allosteric modulator, on acid exposure and symptoms in gastro-oesophageal reflux disease

**DOI:** 10.1136/gut.2008.162040

**Published:** 2009-05-20

**Authors:** C Keywood, M Wakefield, J Tack

**Affiliations:** 1 Addex Pharma, Plan-les-Ouates, Switzerland; 2 Department of Gastroenterology, University of Leuven, Leuven, Belgium

## Abstract

**Background:**

In preclinical models, antagonism of metabotropic glutamate receptor 5 (mGluR5) reduces transient lower oesophageal sphincter relaxations (TLOSRs) and increases LOS pressure. This study evaluated the effect of ADX10059, a potent, selective, negative allosteric modulator of mGluR5, on oesophageal pH-metry and clinical symptoms in GORD.

**Methods:**

Two groups of patients with GORD (n = 12 per group) underwent 24-h oesophageal pH-metry on two sequential treatment days. The patients received oral placebo three times daily (tds) 30 min before a high-fat meal on Day 1 and oral ADX10059 50 mg (Group 1) or 250 mg (Group 2) tds 30 min before a high-fat meal on Day 2. The primary variable was acid exposure (%time pH<4). Secondary variables included number and duration of reflux episodes, number and duration of symptomatic episodes and symptoms recorded in diaries. Comparisons were made for Day 2 (active) versus Day 1 (placebo) treatment and for Group 1 versus Group 2.

**Results:**

ADX10059 250 mg tds significantly decreased the percentage of time with pH<4 from 7.2% to 3.6% (p = 0.01). ADX10059 250 mg tds reduced pH-metry-measured oesophageal acid exposure, throughout the 24 h period, nocturnally and postprandially, and significantly reduced the number and duration of symptomatic reflux episodes (p = 0.03). ADX10059 50 mg tds was not significantly superior to placebo. ADX10059 was generally well tolerated.

**Conclusion:**

The mGluR5 negative allosteric modulator ADX10059 reduced acid reflux which was associated with improvement in clinical symptoms in patients with GORD. ADX10059 appears to have a potential role in the clinical management of GORD.

Proton pump inhibitors (PPIs) are the cornerstone of medical therapy for gastro-oesophageal reflux disease (GORD).^1^,^2^,^3^ However, it has been estimated that up to 30% of patients with GORD remain symptomatic on standard dose (once daily) of PPIs,^4^,^5^,^6^,^7^,^8^ and the majority of these will continue to experience GORD symptoms on even higher doses of PPIs.^4^,^5^,^6^,^7^,^8^ Hence, there is a need for novel therapeutic approaches to GORD.

The most frequent mechanism underlying reflux events is transient lower oesophageal sphincter relaxation (TLOSR), which is an attractive target for the treatment of GORD.^9^ TLOSRs involve a vago-vagal reflex pathway which is activated by gastric distension and integrated in the brain stem to result in relaxation of the lower oesophageal sphincter smooth muscle. A wide variety of transmitters and receptors are expressed centrally and peripherally in the vagal pathway that mediates lower oesophageal sphincter control.^9^,^10^,^11^

Glutamate is the primary neurotransmitter involved in signalling from visceral and somatic primary afferents to the central nervous system.^11^ Anatomical studies of vagal afferents have revealed expression of metabotropic glutamate receptors (mGluRs), including mGluR5, in the nodose ganglia of several species, including humans, and evidence suggests possible localisation in peripheral gastric vagal afferent terminals.^11^ Recent studies in animal models identified selective antagonists of mGluR5 as potent inhibitors of TLOSRs and reflux episodes.^12^,^13^ It has been argued that peripheral mGluR5, expressed in gastro-oesophageal vagal afferent endings, plays a more prominent role in control of TLOSRs as compared with central mGluR5.^10^ These preclinical findings support a role for mGluR5 in the direct control over TLOSRs, providing a mechanistic basis for the clinical development of mGluR5 antagonists for the treatment of GORD.

ADX10059 is a potent selective negative allosteric modulator of the mGluR5 receptor. Rather than acting directly by blocking the glutamate orthosteric binding site, ADX10059 modulates the activity of the mGluR5 receptor by binding to a site distinct from the glutamate binding site (ie, an allosteric site), and diminishes the intra-cellular signal created when glutamate binds to the receptor. The inhibitory effects of a negative allosteric modulator, unlike an orthosteric inhibitor, are non-competitive. Hence, the magnitude and duration of effect of a negative allosteric modulator are not determined solely by its pharmacokinetics. As the negative allosteric modulator acts dynamically with the natural ligand on the receptor function, the effect is more a modulation of physiological responses.

As well as being expressed in the gastrointestinal tract, mGluR5 expression is predominant in areas of the mammalian brain involved in emotional processes, such as the dentate gyrus regions within the hippocampus, regions of the basal ganglia (striatum and nucleus accumbens) and in the dorsal horn of the spinal cord, suggesting a role for these receptors in affective disorders such as anxiety and depression.^14^,^15^ The mGluR5 is also implicated in central pain processing pathways in the trigeminal nucleus caudalis and spinothalamic tract. ADX10059 is also centrally effective, and is additionally being tested in the treatment of migraine. Effects on emotion centres and central pain processing may also be of relevance in the symptomatic treatment of GORD. The present study was a proof-of-concept study aimed at investigating the efficacy, safety and tolerability of the selective mGluR5 antagonist ADX10059 in reducing acid reflux and clinical symptoms in symptomatic patients with GORD.

## Materials and methods

### Study design and objectives

The study was a randomised, single (patient)-blind, placebo-controlled, sequential treatment trial in patients with GORD. The duration of the trial was approximately 4–5 weeks per subject and comprised three visits: screening (Visit 1), two consecutive study treatment days, (placebo followed by active treatment, Visit 2), and a follow-up visit (Visit 3) 1–2 weeks after dosing. As each patient received both placebo and active treatment he/she acted as his/her own control.

The primary objective of the study was to explore the effect of ADX10059 on oesophageal acid exposure measured by 24 h oesophageal pH monitoring. The secondary objectives of the study were: (1) to explore the effect of ADX10059 on diurnal, nocturnal and postprandial episodes of acid reflux; (2) to evaluate the effect of ADX10059 on clinical symptoms of reflux; (3) to evaluate the safety and tolerability of ADX10059 in patients with GORD; and (4) to evaluate the 0–4 h post-dose plasma concentrations of ADX10059 in patients with GORD.

### Conduct of the study

The study was conducted in a single centre (SGS Aster, Paris, France) in an inpatient setting and was performed in accordance with the ethical principles stated in the Declaration of Helsinki as revised by 52nd General Assembly in Edinburgh, 2000, and with the French Huriet law. After Ethics Committee approval, the study was conducted between September and November 2006 in accordance with Good Clinical Practice (GCP) and standard operating procedures (SOP) for clinical investigation and documentation in force at the clinical trial centre.

### Patients

The patients were recruited from a specialist gastroenterology clinic in Paris. All patients had a prior diagnosis of symptomatic GORD made by a gastroenterologist and all had to have a history of good control of heartburn, regurgitation and other GORD symptoms with acid suppressant therapy. Patients who were on acid suppressants at the time of screening had to stop treatment for at least 2 weeks before the study treatment days. Eligible patients were Caucasian men and women aged 18–65 years, weighing between 50 and 100 kg with a body mass index between 18 and 35 kg/m^2^, who were non-smokers or light smokers (<5 cigarettes per day), with normal arterial blood pressure and heart rate.

Patients were excluded if they (1) had any clinically significant acute or chronic disease or significant abnormality in pre-study laboratory tests and physical examination; (2) had received any experimental drug within 30 days prior to screening; (3) were known or suspected alcohol or drug abusers; (4) had undergone surgery or had donated blood within 1 month prior to study start; or (5) had received any drug known to affect hepatic metabolism within 1 month or any drug known to affect renal tubular secretion or gastrointestinal motility, within 2 weeks prior to the first study dose administration. Patients with a history of oesophageal stricture, gastrointestinal bleeding or gastrointestinal surgery were also excluded.

### Procedures

#### Screening and randomisation

Within 3 weeks of the first study treatment day patients attended a screening visit. After patients had provided their written informed consent, the medical histories and demographic data were recorded and safety screening was performed. Eligible patients were randomised to one of two treatment groups: Group 1, placebo (Study Day 1) followed by ADX10059 50 mg tds (Study Day 2); or Group 2, placebo (Study Day 1) followed by ADX10059 250 mg tds (Study Day 2). The choice of doses was based on the pharmacokinetic and tolerability data from a previous repeated dose study in healthy subjects (ref study ADX10059-102, data on file), using the same immediate release formulation; ie, simple drug powder-filled capsules with no excipients.

#### Study drug dosing and pH monitoring days

Patients were admitted to the clinical pharmacology unit on the evening prior to study drug dosing (ie, Day −1). Prior to dosing on Study Days 1 and 2, patients fasted overnight for a minimum of 10 h. Standardised high-fat meals were provided for breakfast, lunch and supper and patients had 30 min to consume each meal. To normalise intake, a fixed amount of water (1500 ml) was supplied and was required to be consumed within each 24 h period. The ambulatory oesophageal pH monitoring was performed using an antimony pH electrode with a separate skin reference electrode (Digitrapper pH100; Medtronic, Tolochenas, Switzerland). The ambulatory pH monitoring unit was calibrated before each use, using standard buffers. The oesophageal pH probe was inserted via one nostril to a distance of approximately 5 cm above the lower oesophageal sphincter. Online continuous pH monitoring was used to locate the position of the lower oesophageal sphincter for each patient.

On Study Days 1 and 2 the probe was inserted and monitoring started about 10 min prior to the first dose administration. The probe was removed after approximately 24 h. Patients had a 30 min pH monitoring-free period, with removal of the catheter, between the two study days so that they could take a shower and change their clothes if they wished.

On each study day, the patients were administered a single oral dose of study medication on three occasions, 30 min before each meal. On Study Day 1 they received placebo, and on Study Day 2 they received ADX10059 50 mg (Group 1) or 250 mg (Group 2). The patients took the capsules with 240 ml of water at room temperature and were dosed while standing. After dosing, the patients remained on their beds, sitting at approximately 45 degrees. The patients were not allowed to lie flat for 4 h following the morning and midday doses, except for study procedures or if clinically indicated.

The timetable of procedures on Study Days 1 and 2 was as follows:

- 07:20 start oesophageal pH recording
- 07:30 study medication dose 1
- 08:00 breakfast
- 12:30 study medication dose 2
- 13:00 lunch
- 19:30 study medication dose 3
- 20:00 dinner
- 22:00 to approx 07:00 bedtime
- 07:00 approx, end of pH monitoring period on Day 1 (on Day 2 end of pH monitoring period was at 07:30)

### Pharmacodynamic efficacy measures

#### 24 h oesophageal pH measurement

Oesophageal pH was recorded for approximately 24 h starting on Study Day 1 and Study Day 2. pH measurements were captured every 4 s resulting in approximately 21 600 measurements for each 24 h period. Each variable was calculated for the 24 h recording period and for the upright diurnal period (07:30 to 22:00) and the supine nocturnal period (22:00 to 07:30 approx). The percentage time for oesophageal pH<4 was calculated from the continuous online monitoring.

#### Number and duration of reflux episodes

The number and total duration of gastro-oesophageal reflux episodes was recorded. In accordance with the standards of the clinical pharmacology unit, a reflux episode was defined as seven consecutive measures with a pH<4; ie, at least 28 s. The total duration of reflux episodes was the sum of all actual times of reflux episodes ⩾28 s. The number of gastro-oesophageal reflux episodes and the total duration of time with gastro-oesophageal reflux episodes was summarised for the 24 h, diurnal and nocturnal periods. Oesophageal acid clearance was expressed as the mean duration of acid reflux events.

#### Postprandial reflux episodes

The postprandial periods were defined as the period of 4 h following each meal; ie, from 08:00 to 12:00, 13:00 to 17:00 and 20:00 to 24:00. Postprandial reflux episodes were documented by a pH drop to <4 for at least 28 s and as food has an effect on neutralising stomach acid, pH drops ⩾1 for at least 28 s were also used to measure postprandial reflux. The number and duration of postprandial reflux events were summarised for each treatment. In addition, the number and total duration of pH drops ⩾1 were summarised for the 24 h and nocturnal periods.

#### Clinical symptoms of reflux

Patients recorded the occurrence and duration of symptomatic reflux episodes in a diary on each treatment day. Patients were asked to note when they experienced typical GORD symptoms. Heartburn and regurgitation were not evaluated separately. The number and duration of symptomatic reflux events were summarised for the 24 h period.

### Safety and pharmacokinetic measures

Safety assessments were made at screening, at follow-up and at regular time points during the study drug administration days. The safety measures comprised full physical examination, urinalysis, pregnancy testing (screening and follow-up only), heart rate, blood pressure, haematology, biochemistry, 12-lead ECG and regular adverse events enquiry.

Blood samples for plasma concentrations of ADX10059 were taken on both study days (to maintain the blinding to the patient) pre-dose and at 0.5, 1.0, 2.0, 3.0 and 4.0 h after each dose. From the plasma concentration versus time profiles the following pharmacokinetic parameters were assessed: t_max_, C_max_, AUC_0–4_ and AUC_0–∞_.

### Statistical methods

The primary efficacy variable was the percentage of time with oesophageal pH<4 comparing ADX10059 with placebo. Secondary variables included: (1) the percentage of time with oesophageal pH<4 in the nocturnal and diurnal periods; (2) the number and duration of reflux episodes (oesophageal pH<4) during the 24 h, nocturnal, and the 4 h postprandial periods; (3) the number and total duration of pH drop ⩾1, during the 24 h, nocturnal and the 4 h postprandial periods; (4) oesophageal acid clearance; and (5) the number and total duration of symptomatic episodes of GORD.

The analysis populations were as follows. The safety population included all randomised patients, who received the study drug and had post-dosing data. The pharmacodynamic population included all patients who completed the study without major protocol violations or events implying a bias for pharmacokinetic evaluation and with two complete pH-metry profiles (Study Days 1 and 2).

Intra-individual comparison between placebo and active drug was performed during the two successive assessments. Statistical analysis for efficacy was performed on the pharmacodynamic population. For the primary and secondary endpoints, all parameters were analysed on the change from baseline (placebo day 1 value) by analysis of covariance (ANCOVA) using dose level as a fixed effect and baseline as covariate. Estimates (least squares means) of dose effects and differences between doses were provided with their respective 95% confidence intervals. Quantitative parameters were described per group, dose level and time point using n (number of observations), mean, median, standard deviation (SD), minimum, and maximum. The 95% confidence interval of the mean was included for changes from baseline. All statistical tests were two-tailed and the significance threshold was set at the 5% level.

This was an exploratory study without a formal statistical sample size calculation. A total of 12 patients per dose group was deemed to be sufficient to obtain meaningful data on the pharmacodynamic effect of ADX10059 on 24 h pH and clinical symptoms in this proof-of-concept study.

## Results

### Patient demographics

**Figure 1:**
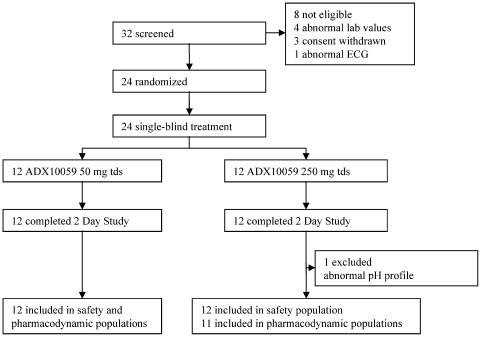
Patient disposition.

Thirty-two patients were screened, of which 24 were randomised (eight were not eligible, four had abnormal laboratory values, three withdrew consent and one had an abnormal ECG). All 24 randomised patients (12 in Group 1 comparing ADX10059 50 mg to placebo, and 12 in Group 2 comparing ADX10059 250 mg to placebo) completed the study and were included in the safety and pharmacokinetic analyses. One male patient in Group 2 was excluded from the pharmacodynamic population due to missing pH-metry data on Study Day 1 when his pH probe became displaced. Subject disposition is shown in fig 1. The treatment groups had similar demographic and baseline characteristics (table 1).

**Table 1:** Demographic characteristics of the patients

Characteristic	Group 1 (n = 12)	Group 2 (n = 12)
Race (Caucasian)	12 (100%)	12 (100%)
Sex (female)	3 (25%)	2 (16.7%)
Age (years)	44.6	45.1
Weight (kg)	77.9	79.8
Body mass index (kg/m^2^)	26.1	26.2
Light smokers (<5 cigarettes/day)	3 (25.0%)	2 (16.7%)
Previous GORD medications	6 (50%)	10 (83.3%)

GORD, gastro-oesophageal reflux disease.

The majority of the patients were men (nine in Group 1 and 10 in Group 2) with an average age of approximately 45 years. In Group 1 50% of patients and in Group 2 83% of patients, were previously using regular acid suppression therapy.

### Primary efficacy: percentage of time pH<4 in 24 h

At baseline, patients in Group 1 tended to have a greater percentage of time pH<4 in the 24 h period, but this difference was not significant (14.9 (SD 13.9)% Group 1; 7.2 (SD 5.8)% Group 2, p  =  NS). There was no significant effect of ADX10059 50 mg tds on percentage of time pH<4 (table 2). ADX10059 250 mg tds significantly decreased the percentage of time that pH was <4 in the 24 h period to 3.6 (SD 3.2)% (p = 0.0144) and in the nocturnal period from 9.7 (SD 10.2)% to 3.7 (SD 6.0)% (p = 0.0028).

**Table 2:** Percentage time when the pH was less than 4

Treatment group	Percentage time pH<4 in 24 h	Percentage time pH<4, diurnal	Percentage time pH<4, nocturnal
**Group 1, n = 12**			
Placebo	14.9	9.5	22.7
ADX10059, 50 mg	15.1	12.8	18.9
Estimate change from baseline	2.71	4.75	0.19
95% Confidence interval	−2.05 to 7.48	0.14 to 9.37	−5.88 to 6.26
p Value	NS	0.0442*	NS
			
**Group 2, n = 11**			
Placebo	7.2	5.2	9.7
ADX10059, 250 mg	3.6	3.4	3.7
Estimate change from baseline	−6.41	−3.41	−10.37
95% Confidence interval	−11.4 to −1.42	−8.24 to 1.42	−16.73 to −4.01
p Value	0.0144	NS	0.0028

*Increased compared with placebo.

NS, not significant.

### Secondary efficacy measures

#### Number and duration of acid reflux episodes: 24 h and nocturnal periods

At baseline, there were no significant differences between the treatment groups for either the number or total duration of gastro-oesophageal acid reflux episodes during the 24 h period.

Compared with placebo, ADX10059 50 mg t.i.d. in Group 1 did not significantly alter the mean number of acid reflux episodes or the total duration of episodes, in all time periods (table 3). The average oesophageal acid clearance was not altered by ADX10059 50 mg tds (3.9 (SD 0.4) vs 3.4 (SD 0.4) min; NS).

**Table 3:** Group 1 (ADX10059 50 mg tds): number and duration of reflux episodes and clinical symptoms

Efficacy variable	ADX10059 50 mg tds, (n = 12)	Placebo tds, (n = 12)	p Value
Mean (SD) number reflux episodes (pH<4) in 24 h	65.3 (48.9)	51.9 (43.3)	NS
Mean (SD) total duration of reflux episodes (pH<4) in 24 h (min)	185.3 (136.4)	184 (172.6)	NS
Mean (SD) number nocturnal reflux episodes (pH<4)	28.6 (26.9)	21.4 (20.7)	NS
Mean (SD) total duration of reflux episodes (pH<4) nocturnal period (min)	97.1 (76.6)	118 (113.3)	NS
Mean (SD) number of symptomatic episodes	5.3 (3.3)	6.6 (4.6)	NS
Mean (SD) duration of symptomatic episodes (min)	28.7 (43.9)	43.2 (81.0)	NS

NS, not significant; tds, three times daily.

**Figure 2:**
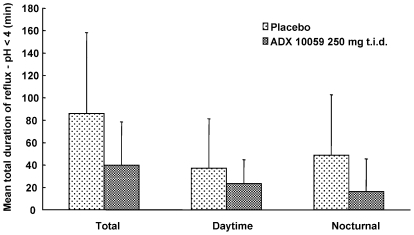
Mean total duration of reflux episodes.

In Group 2, ADX10059 250 mg tds significantly decreased the mean total duration of acid reflux episodes during the 24 h period (mean 40 (SD 39) vs placebo 86 (SD 72) min p = 0.0132) and during the nocturnal period (mean 16 (SD 29) vs placebo 49 (SD 54) min, p = 0.0021) (fig 2). There was also a trend towards a decrease in the number of episodes of acid reflux at all time points, but the differences were not statistically significant (table 4). The average oesophageal acid clearance was not significantly altered by ADX10059 250 mg tds (3.0 (SD 0.3) vs 2.4 (SD 0.3) min, NS).

**Table 4:** Group 2 (ADX10059 250 mg tds): number and total duration of reflux episodes and clinical symptoms

Efficacy variable	ADX10059 250 mg tds, (n = 11)	Placebo tds, (n = 11)	p Value
Mean (SD) number reflux episodes (pH<4) in 24 h	20.5 (19.4)	32.7 (20.3)	NS
Mean (SD) total duration of reflux episodes (pH<4) in 24 h (min)	39.9 (38.7)	86 (72.2)	0.0132
Mean (SD) number nocturnal reflux episodes (pH<4)	6.4 (9.9)	13.6 (12.3)	NS
Mean (SD) total duration of reflux episodes (pH<4) nocturnal period (min)	16.4 (29)	48.7 (54)	0.0021
Mean (SD) number of symptomatic episodes	1.9 (3.8)	7.0 (13.8)	0.031
Mean (SD) duration of symptomatic episodes (min)	5.2 (12.6)	13.9 (20.1)	0.031

tds, three times daily.

#### Total duration pH drops ⩾1: 24 h and nocturnal

Overall, in Group 2 there was a reduction in the total duration of pH drop ⩾1 in 24 h, from a mean of 118 (SD 57) min with placebo to a mean of 75 (SD 46) min during active treatment (p = 0.054) of which the nocturnal duration significantly decreased from a mean of 58 (SD 42) min to a mean of 31 (SD 34) min (p = 0.0049).

In Group 1, the 50 mg dose of ADX10059 did not significantly alter the number or duration of pH drops ⩾1 in the postprandial periods, the nocturnal or the 24 h period.

#### Postprandial reflux

In Group 1, the 50 mg dose of ADX10059 did not significantly alter the number or duration of reflux episodes using either oesophageal pH<4, or pH drops ⩾1, in the 4 h postprandial periods.

Using pH drops ⩾1, in Group 2 ADX10059 250 mg tds significantly decreased either the number or duration of reflux episodes in the postprandial periods.

**Figure 3:**
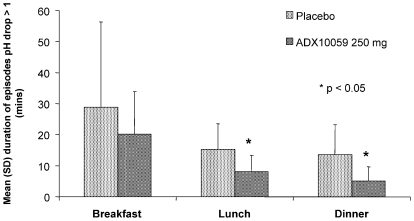
Mean duration of postprandial episodes of pH drop >1.

The number of drops of pH⩾1 significantly decreased in the post-breakfast period (mean 6.8 (SD 5.4) vs placebo 9.7 (SD 4.0), p = 0.041). Post-lunch and post-dinner the differences in the number of episodes of pH drops ⩾1 were not statistically significant. The duration of pH drops ⩾1 significantly decreased in the post-lunch period (mean 8.1 (SD 5.3) vs placebo 15 (SD 8.3) min, p = 0.0371) and post-dinner period (mean 5.1 (SD 4.6) vs placebo 13.5 (SD 9.7) min, p = 0.0146) (fig 3).

In Group 2, using oesophageal pH<4 to determine postprandial reflux, ADX10059 showed a numerical reduction in the number and duration of episodes but none achieved statistical significance.

#### Clinical symptoms of reflux

The mean number of patients who reported symptomatic reflux episodes was significantly lower than the total number of reflux episodes detected by pH monitoring.

ADX10059 250 mg tds resulted in a statistically significant reduction in the number and duration of symptomatic reflux episodes (table 4). The number of episodes was reduced from 7 (SD 13.8) on the placebo baseline day to 1.9 (SD 3.8) on the active treatment day (p = 0.031) and the mean total duration of symptomatic reflux was reduced from 13.9 (SD 20.1) to 5.2 (SD 12.6) min (p = 0.031) In the ADX10059 50 mg group, the number of symptomatic episodes was not significantly reduced (table 3).

### Pharmacokinetics

**Figure 4:**
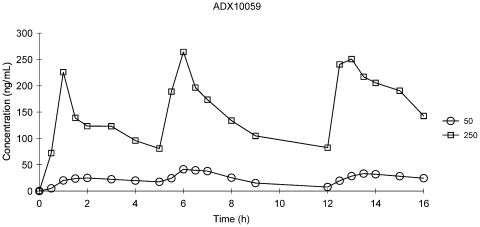
Mean plasma concentration versus time profiles following repeated oral doses three times daily of 50 mg or 250 mg of ADX10059 for 1 day (linear scale).

The mean plasma concentration–time curves are shown in fig 4. Following oral administration, ADX10059 was rapidly absorbed and was detectable in plasma 30 min after dosing in the majority of patients. There was inter-individual variability in plasma exposure (coefficient of variation of approx 50% for both doses for AUC_0–16_). For ADX10059 50 mg, the geometric mean C_max_ ranged from 27.3 ng/ml after dose 1 to 35.4 ng/ml after dose 3; and for ADX10059 250 mg, ranged from 221 ng/ml after dose 1 to 283 ng/ml after dose 3. The time to reach maximum plasma concentration was variable and ranged between 0.5 and 4 h regardless of dose and administration number.

Correlations of plasma concentration with reflux episode duration in the whole 24 h period and in each of the postprandial periods were performed. Although the drug effect seems to increase with increasing ADX10059 dose, no clear pharmacodynamic/pharmacokinetic relationship could be seen.

### Safety and tolerability

ADX10059 given as three doses in 1 day was generally well tolerated by the patients with GORD. No serious adverse events were reported. One patient in Group 1 reported flatulence after receiving placebo. The incidence of adverse events was higher in Group 2, the 250 mg tds group, 11/12 (91.6%) than in the 50 mg t.i.d. group 2/12 (16.7%). In Group 1, somnolence, cough and rhinorrhoea were reported in 1/12 patients (8%). In Group 2, the most commonly reported adverse events were related to the central nervous system and the most common single adverse event was dizziness (9/12 patients, 75%). The dizziness was accompanied by nausea in 4/12 (33%) of the patients. In addition, 2 (17%) patients reported dysuria and other events occurring in 1/12 (8%) patients were tinnitus, visual accommodation disorder, dry mouth, vomiting, paresthesia, hypoesthaesia, feeling drunk and hot flush. All adverse events except one occurred following the first or second dose; none of the events was described as severe and all resolved without sequelae. No clinically significant changes in safety monitoring parameters for haematology, blood chemistry, urinalysis, vital signs, physical examination or 12-lead ECG were reported.

## Discussion

Inhibition of mGluR5 has been shown to reduce transient lower oesophageal sphincter relaxation episodes and increase lower oesophageal sphincter tone in animals,^12^,^13^ a mechanism which could have application in the prevention of gastro-oesophageal reflux in humans. To our knowledge, the effect of mGluR5 inhibition on TLOSRs and reflux events has not been reported in humans. The aim of the present study was to assess the effect of ADX10059 on gastro-oesophageal reflux in patients with GORD, using pH-metry to detect episodes of acid reflux and hence indirectly study LOS function.

To this end, 24 patients with GORD diagnosed in a specialist gastroenterology clinic were randomised in two groups of 12 patients (Groups 1 and 2). In each group, the effect of ADX10059 (50 mg tds or 250 mg tds for 1 day) was compared with placebo. The doses of ADX10059 were selected based upon the pharmacokinetic and tolerability data from a previous repeated dose study in healthy subjects (ref study ADX10059-102 data on file). The upper and lower extremes of the doses from that study were chosen for this study to explore the safety, tolerability and pharmacodynamics across a dose range that might have therapeutic potential in patients with GORD. In both groups, each patient received placebo treatment on Day 1 and then ADX10059 on Day 2 in a single-blind fashion, and therefore acted as his/her own control. Patients were blinded as to the treatment sequence so that they could objectively evaluate their clinical symptoms on each treatment day.

Consistent with previous results obtained with the immediate release, powder-filled capsule, ADX10059 was quite rapidly absorbed with a median plasma peak occurring between 1.0 and 2.0 h following administration. As already observed in healthy subjects, there was a large inter-individual variability for plasma concentrations and pharmacokinetic parameters. No clear relationship was drawn from the review of drug exposure and drug effect. The safety and tolerability profile was also consistent with that observed at these doses in previous single and repeated dose studies of ADX10059 in healthy subjects (refs. Data on file, studies ADX10059-101, 102 and 103). The central nervous system effects (eg, dizziness) seen in the 250 mg tds group are consistent with the mechanism of action and the rapid absorption following dosing using the immediate release capsule. The side effect profile in the higher dose group is considered undesirable for long-term treatment of GORD, therefore a modified release formulation which is less rapidly absorbed and which has been shown to reduce the occurrence of central nervous system side effects (data on file Study ADX10059-104), has been developed and will be used for subsequent studies. While the 50 mg of ADX10059 tds had no statistically significant effect, the 250 mg dose of ADX10059 tds produced significant improvement in pH-metry-derived reflux and in the symptomatic expression of GORD. To our knowledge, this is the first study in humans to support the findings of previous animal studies on the effect of mGluR5 antagonism on reflux events. Hence the concept that the mGluR5 negative allosteric modulator (NAM) may reduce reflux and have therapeutic potential in GORD was supported by the findings of this study.

Based on animal studies with other mGluR5 NAMs, 3-[(2-methyl-1,3-thiazol-4-yl)ethynyl]pyridine (MTEP) and 2-methyl-6-phenylethynylpyridine (MPEP), the effects of ADX10059 on oesophageal acid exposure are expected to reflect inhibition of TLOSRs through a peripheral mode of action.^10^ The decrease in acid reflux events in the postprandial period, when TLOSRs occur most frequently,^9^ is compatible with such a mechanism of action. It is less clear whether the inhibition of nocturnal reflux events is also attributable to inhibition of TLOSRs, or whether other factors, such as an increase in resting LOS pressure which was apparent from animal studies using MTEP and MPEP,^12^ may play an additional role. Inhibition of nocturnal reflux has also been observed with baclofen, which inhibits TLOSRs through γ-amino butyric acid type B receptor agonism.^9^,^16^,^17^ Although the 250 mg dose of ADX10059 significantly decreased the total duration of acid reflux events, this was not associated with a significant decrease of the number of reflux events for the 24 h measurement period. This observation suggests shortening of reflux episodes, which could be due to improved oesophageal clearance (although this was not observed in this study) or to a smaller volume of refluxed material during reflux events. Elucidating the mechanisms underlying the anti-reflux effects of ADX10059 will require additional studies.

This study was an initial exploratory study and as such the authors recognise that there are features of the design which could potentially impact the interpretation of the results and for which the rationale should be explained. A single-blind sequential day dosing regimen was chosen principally for logistic reasons, so that the patients would not have to undergo pH monitoring for an extended duration or have to undergo repeated admissions to the unit, which would have been required if the study had been a randomised cross-over design. The patients were blinded to the treatment sequence and underwent exactly the same procedures on Study Days 1 and 2 (including blood sampling for pharmacokinetics) in order to minimise any effect on subjective symptom reporting. As the majority of evaluations were objective physiological measures, the single-blind design should not affect these. However, it is possible that the measures on Study Day 2 could differ to those on Study Day 1 due to the study conditions and it would normally be preferable to randomise the treatment order to mitigate this. Overall, using the 250 mg dose, significant decreases in acid reflux parameters and in reflux-related symptoms were observed. Although oesophageal pH monitoring shows considerable intra-individual day-to-day variability, systematic order effects with lower acid exposures on Study Day 2 are not found.^18^,^19^,^20^,^21^,^22^,^23^,^24^,^25^,^26^,^27^ Also, the consistent effects in the 250 mg dose group were not seen in the 50 mg dose group suggesting a dose response effect. Hence the study design is not considered to significantly impact the overall interpretation of the results.

The definition of reflux events as seven consecutive episodes of oesophageal pH<4 was one that was standard for the clinical pharmacology unit. It is recognised that this may lead to under-reporting of the number of reflux events and only acid reflux events can be captured in this way. The total percentage of pH<4 was derived from the continuous pH monitoring and so reflux events that were less than 28 s were captured in this measurement. Furthermore, as food may neutralise the stomach pH, drops in pH of ⩾1 for ⩾28 s were used, in addition to the measure of pH<4 for ⩾28 s, to more accurately identify postprandial reflux events. Impedance pH monitoring is a more sensitive measure of reflux events capturing all types of reflux event and this will be used for subsequent studies.

## Conclusions

To our knowledge, this is the first study to report on the effects of a mGluR5 NAM in patients with GORD. ADX10059 250 mg tds reduced acid reflux as measured by pH-metry, and this was associated with improvement in clinical symptoms. The study confirms the potential for the mGluR5 NAM ADX10059 in the treatment of GORD. Potential therapeutic applications to be evaluated include add-on therapy in patients with GORD with incomplete response to PPIs, or monotherapy in those for whom PPIs are unsuitable.
